# Evaluation of erectile function after anastomotic vs substitutional urethroplasty for bulbar urethral stricture

**DOI:** 10.1080/2090598X.2020.1805965

**Published:** 2020-08-18

**Authors:** Rabea G. Omar, Mostafa M. Khalil, Hesham Alezaby, Ahmed Sebaey, Hammouda Sherif, Ahmed Mohey

**Affiliations:** Department of Urology, Faculty of Medicine, Benha University, Benha, Egypt

**Keywords:** Stricture urethra, anastomotic urethroplasty, substitution urethroplasty, international index of erectile function-15

## Abstract

**Objective:**

To evaluate erectile function (EF) after anastomotic (AU) vs substitutional urethroplasty (SU) for bulbar urethral stricture.

**Patients and methods:**

This was a prospective comparative non-randomised clinical study conducted in the Department of Urology, Benha University Hospital, between September 2015 and September 2018, involving 34 male patients with urethral stricture. Preoperatively, all the patients completed the International Index of Erectile Function (IIEF)-15 (EF Domain) to establish baseline sexual function. AU was performed in 21 patients and SU in 13. The IIEF-15 (EF Domain) was administered again at 3- and 6-months postoperatively and compared to the preoperative results.

**Results:**

A total of 34 patients were included in the study, 21 in the AU group with a stricture length of ≤3 cm, and 13 in the SU group with a stricture length of >3 cm. All patients were sexually active preoperatively. In the AU group, seven patients developed erectile dysfunction (ED) at 3-months postoperatively, with four of them improving during the subsequent 3 months, but three had persistent ED at 6-months postoperatively. In the SU group, two patients developed ED at 3-months postoperatively and they improved during the subsequent 3 months. In the AU group, the mean IIEF-15 (EF Domain) score was 27.6 preoperatively, which decreased to 25.6 at 6-months postoperatively; however, this was statistically insignificant (*P* = 0.10). While in the SU group, the mean IIEF-15 (EF Domain) score was the same before and at 6 months after SU at 27.2 (*P* = 1.0).

**Conclusion:**

At 6-months postoperatively, there was no statistically significant impact of urethroplasty for bulbar urethral stricture on erectile function.

**Abbreviations**: AU: anastomotic urethroplasty; ED: erectile dysfunction; EF: erectile function; IIEF: International Index of Erectile Function; SU: substitutional urethroplasty

## Introduction

Urethral stricture is considered a surgical challenge amongst urologists and various treatment options are available including: direct internal urethrotomy, urethral stents, and urethroplasty either anastomotic (AU) or substitutional (SU) [1].

The AU technique is only suitable for short segment strictures of ≤3 cm, which results in tension-free approximation, while SU urethroplasty is suitable for longer stricture using either flaps or grafts to reconstruct the diseased urethra [2].

Most of the previously published studies have focussed on recurrence of urethral stricture and the incidence of incontinence [3], while few of them have addressed sexual satisfaction after urethroplasty, particularly erectile function (EF), as the most important factor for sexual satisfaction [4].

In previous studies, few have compared the impact of AU vs SU on sexual function and that have used the International Index of Erectile Function (IIEF)-5 score, while in the present study we evaluated the EF after AU vs SU for bulbar urethral stricture using the IIEF-15 (EF Domain) score.

## Patients and methods

This was a prospective comparative non-randomised clinical study conducted in the Department of Urology, Benha University Hospital, between September 2015 and September 2018, involving 34 male patients with urethral strictures. Patients with erectile dysfunction (ED) and patients with recurrent urethral stricture were excluded from the study.

All patients were evaluated by: urological history concerning history of trauma regarding genitalia and pelvis fracture; iatrogenic causes, such as cystoscopy or long-term catheter; and history of urethritis or gonorrhoea. A detailed medical and sexual history, using the validated International Index for Erectile Function IIEF-15 (EF Domain) questionnaire, was also taken. A general examination and genitourinary system examination, such as palpation of the urethra for induration, was performed; and routine preoperative laboratory investigations, ascending and voiding cystourethrogram, and urethral ultrasonography (US).

All enrolled patients completed the IIEF-15 (EF Domain) preoperatively to establish baseline sexual function.

The IIEF-15 is used worldwide for evaluation of male EF and is divided into five domains of sexual function including: EF, orgasmic function, satisfaction, sexual desire, and overall satisfaction

Each domain is scored and the range of the total score is from 5 to 75. As regard the EF Domain, lower scores mean worse ED, while higher scores mean less ED [5]. The EF Domain score is divided into four groups: Group I: score 1–10, severe ED; Group II: score 11–16, moderate ED; Group III: score 17–25, mild ED; Group IV: score 26–30, no ED [6].

### Surgical techniques

**Figure 1.**
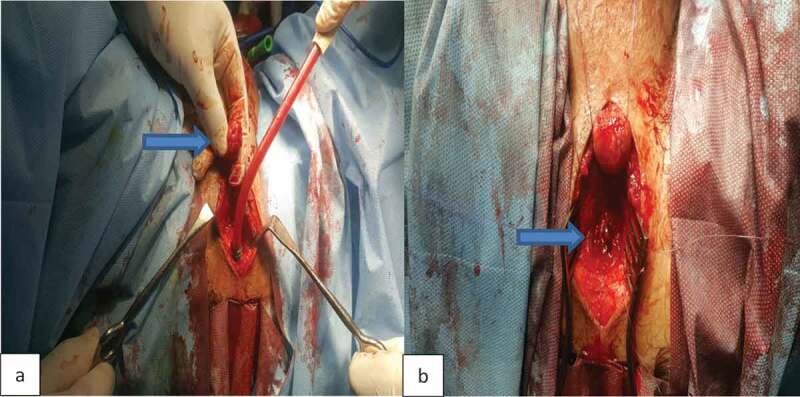
Bulbo-membranous stricture. (a) Distal end of the stricture (arrow). (b) Proximal end of the stricture with metal dilator inserted antegrade (arrow)

**Figure 2.**
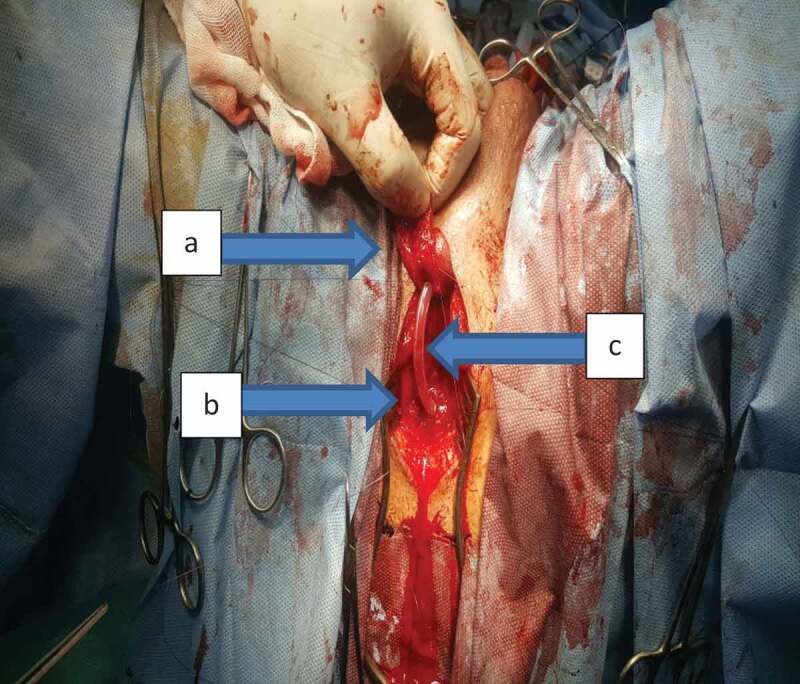
End-to-end anastomosis of bulbo-membranous stricture. (a) Distal segment. (b) Proximal segment. (c) Urethral catheter

**Figure 3.**
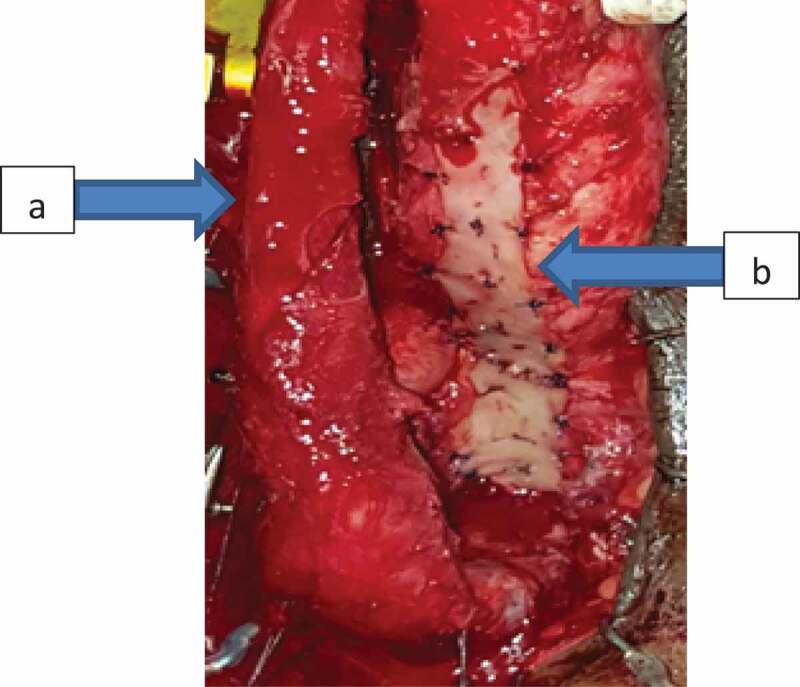
Buccal mucosal graft urethroplasty of bulbo-penile urethral stricture. (a) Corpus spongiosum with its bulbar urethra. (b) Buccal mucosal graft fixed at the under surface of the corpus cavernosum and the deep perineal membrane

**Figure 4.**
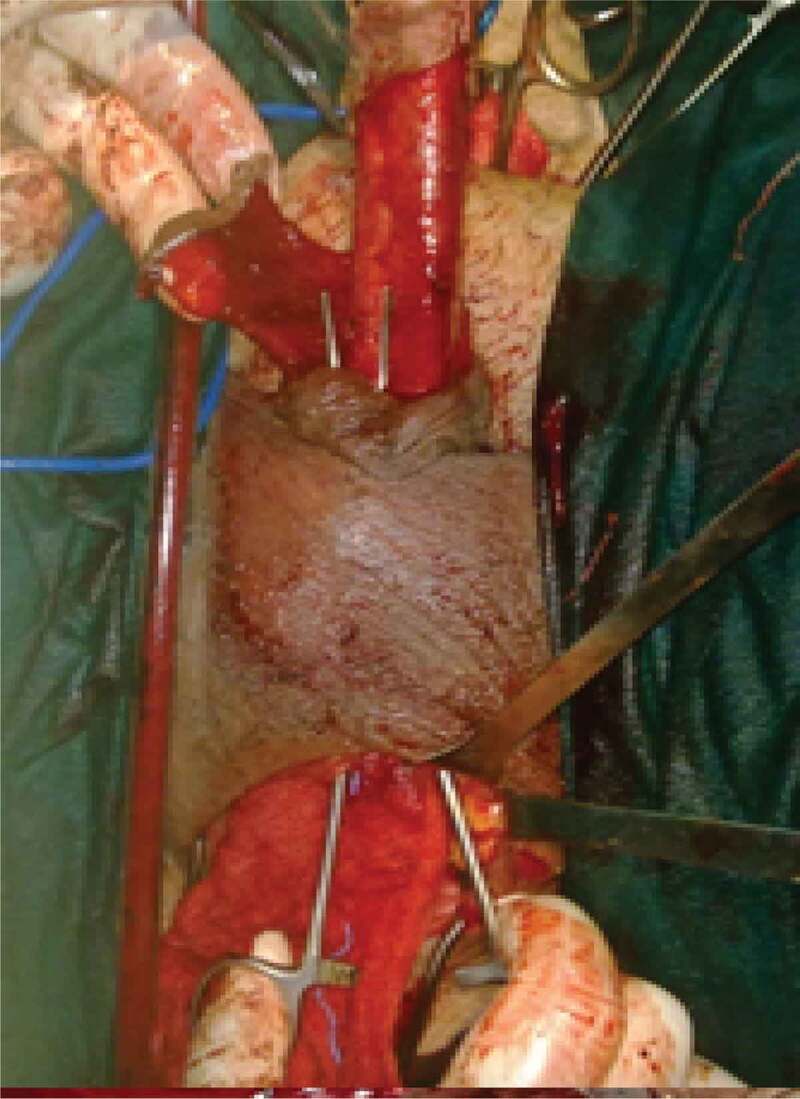
McAninch penile skin-flap urethroplasty for long segment bulbar urethral stricture

A total of 34 patients were included in our study. According to preoperative assessment (measurement of the stricture length by ascending and voiding cystourethrogram and urethral US), and intraoperative measurement of the stricture using a ruler, all patients were managed either by AU or SU. AU was indicated for strictures of ≤3 cm and was performed in 21 patients; where excision of the stricture segment and spatulation of both ends and a tension-free anastomosis was made (Figures 1 and 2). SU was indicated for strictures of >3 cm and was performed in 13 patients. Two subtypes of SU were performed: a) buccal mucosal graft (BMG) used in eight patients, where stricturotomy was done at the 12 o’clock position extending to normal urethral lumen at both ends measuring the length of this incision and harvesting an equal length of the buccal mucosa to augment the urethra (Figure 3); b) penile skin flap used in five patients, where stricturotomy was done at the 12 o’clock position extending to normal urethral lumen at both ends measuring the length of this incision, a dorsal transverse penile skin flap (McAninch flap) was created to augment the urethra at this site (Figure 4).

### Follow-up

Postoperative antibiotics, NSAIDS and anti-cholinergics were given when indicated, and the urethral catheter was removed after 4 weeks, and ascending and voiding cystourethrogram was done. At 3-months postoperatively: ascending and voiding cystourethrogram was conducted to exclude recurrence of urethral stricture, and the IIEF-15 (EF Domain) was repeated for comparison with the preoperative results. At 6-months postoperatively the IIEF-15 (EF Domain) was again administered for comparison with the results preoperatively and at 3-months postoperatively, to evaluate the effect of different methods of bulbar urethroplasty on postoperative EF. Penile Doppler US was done when indicated. None of the patients received phosphodiesterase type 5 inhibitors in either group.

The collected data were tabulated and analysed using the Statistical Package for the Social Sciences (SPSS®), version 16 (SPSS Inc., Chicago, IL, USA). Categorical data were presented as number and percentages, while quantitative data were expressed as mean ± standard deviation (SD) and range.

## Results

Overall, 34 patients were included in the study (21 patients in the AU group and 13 in the SU group); the detailed characteristics of all patients and the types of urethroplasty performed are summarised in Table 1. The mean (SD, range) age of the patients in the AU group was 33.2 (12.1, 21–60) years and in the SU group was 47.8 (9.1, 30–65) years, with no statistically significant difference. The mean (SD, range) stricture length in AU group was 2.04 (0.5, 1.5–3) cm and in the SU group was 4.3 (0.9, 3.5–6) cm, which was statistically significantly different (*P* = 0.005). The site of the stricture in the AU group was proximal bulbar in 10 patents (47.6%) and mid-bulbar in 11 (52.4%), while in the SU group eight of the 13 were mid-bulbar and five were distal bulbar, which was statistically significantly different (*P* = 0.034). The cause of stricture in AU group was traumatic in all cases (100%), while in the SU group inflammation was the cause of stricture in most of the patients in 10/13 and traumatic in three, which was statistically significantly different (*P* = 0.027).

**Table 1.** Patients’ baseline characteristics

Characteristic	AU group	SU group	*P*
Number of patients	21	13	
Age, years, mean (SD, range)	33.2 (12.1, 21–60)	47.8 (9.1, 30–65)	0.2
Stricture length, cm, mean (SD, range)	2.04 (0.5, 1.5–3)	4.3 (0.9, 3.5–6)	0.005
Site of stricture, *n* (%) or *n/N* Proximal bulbar Mid bulbar Distal bulbar	10 (47.6) 11 (52.4) 0 (0)	0/13 8/13 5/13	0.034
Cause of stricture, *n* (%) or *n/N* Inflammatory Traumatic	0 (0) 21 (100)	10/13 3/13	0.027
Preoperative ED, *n* (%) or *n/N* No ED ED	21 (100) 0 (0)	13/13 0/13	0.9

No patients had ED preoperatively in either group. In the AU group, at 3-months postoperatively seven patients developed ED (two of them had recurrent urethral strictures and visual internal urethrotomy was done) and at the 6-month follow-up four had recovered and became sexually active, but the remaining three patients have persistent ED (including the two patients with recurrent stricture) and confirmed by penile duplex US. In the SU group, at the 3-month follow-up two patients had developed ED and at the 6-month follow-up these two patients recovered and became sexually active. There was no significant statistical difference between the groups as shown in Table 2.

**Table 2.** Assessment of EF and mean IIEF-15 score over the period of the study among the studied groups

	AU group	SU group	*P*
ED, *n* (%) or *n/N*			
Preoperative ED No ED	0/21 (0) 21(100)	0/13 13/13	-
3-months postoperatively ED No ED	7 (33.3) 14 (66.7)	2/13 11/13	0.42
6-months postoperatively ED No ED	3 (14.3) 18 (85.7)	0/13 13/13	0.27
IIEF-15 (EF Domain) score, mean (SD)			
Preoperative	27.6 (1.32)	27.2 (1.64)	0.29
3-months postoperatively	24.4 (5.6)	26.6 (2.59)	0.60
6-months postoperatively	25.6 (5.3)	27.2 (1.64)	0.87
*P*	0.005	0.13	

**Figure 5.**
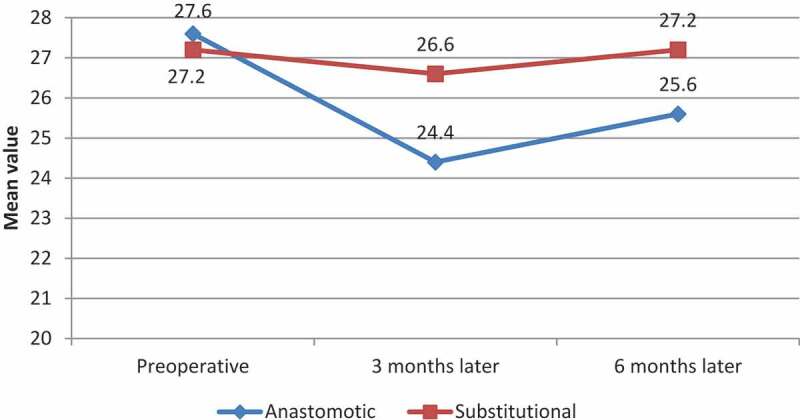
The IIEF-15 (EF Domain) scores over the study period

In the AU group, there was a statistically significant difference between the mean IIEF-15 (EF Domain) score preoperatively, and at the 3- and at 6-month follow-ups (*P* = 0.005); while in the SU group, there was no statistically significant difference between the mean IIEF-15 score preoperatively, and at the 3- and 6-month follow-ups (*P* = 0.13). When comparing both groups regarding the change in the mean IIEF-15 (EF Domain) score over the period of the study, there was no statistically significant difference, as shown in Table 2 and Figure 5.

When comparing the preoperative IIEF-15 score with that at the 3-month follow-up, we found that in the AU group the mean IIEF-15 score was statistically significantly lower at 3 months (*P* = 0.015), while in the SU group, although the mean IIEF-15 score was also lower at the 3-month follow-up it was statistically non-significant (*P* = 0.18), as shown in Table 3.

**Table 3.** Comparison of the preoperative IIEF-15 (EF Domain) score with the score at 3-months postoperatively in the AU and SU groups

	IIEF-15 (EF Domain) score			
	Preoperative	3-months postoperatively	Wilcoxon test	*P*
AU group, mean (SD, range)	27.6 (1.32, 26–30)	24.4 (5.6, 9–30)	2.39	0.015
SU group, mean (SD, range)	27.2 (1.64, 26–30)	26.6 (2.59, 21–30)	1.34	0.18

When comparing the preoperative IIEF-15 score with that at the 6-month follow-up, we found that in the AU group the mean IIEF-15 score was again lower than preoperatively, but was statistically non-significant (*P* = 0.10), while in the SU group the mean IIEF-15 score was the same at 6-months postoperatively as preoperatively (*P*= 1.0), as shown in Table 4.

**Table 4.** Comparison of the preoperative IIEF-15 (EF Domain) score with the score at 6-months postoperatively in the AU and SU groups

	IIEF-15 (EF Domain) score			
	Preoperative	6-months postoperatively	Wilcoxon test	*P*
AU group, mean (SD, range)	27.6 (1.32, 26–30)	25.6 (5.3, 9–30)	1.63	0.10
SU group, mean (SD, range)	27.2 (1.64, 26–30)	27.2 (1.64, 26–30)	0.0	1.0

Thus, at 6-months postoperatively, there was no statistically significant impact of urethroplasty for bulbar urethral stricture on EF.

## Discussion

Urethroplasty is considered the ‘gold standard’ surgical treatment for urethral stricture, with a success rate of >90% and a low incidence of significant complications [7], but one of the significant complications is ED that ranges from 0% to 40% [8].

Anger *et al*. [9] assessed EF after urethroplasty, using the IIEF-5 questionnaire in a study of 25 men with bulbar urethral stricture treated with bulbar urethroplasty. They found that, there was no effect of bulbar urethroplasty on EF at 6-months postoperatively in comparison with the preoperative data. Erickson *et al*. [10] also performed a prospective study of 52 men with bulbar urethral stricture who underwent urethroplasty. They found that 20 patients developed ED in the postoperative period and at 6.3-months follow-up, 18 of them recovered EF, and they concluded that there was no significant incidence of ED after bulbar urethroplasty.

Dogra *et al*. [11] suggested that 20% of patients with bulbar urethral stricture had ED after urethroplasty and it has been shown that bulbar urethral stricture negatively affects EF after surgery. A meta-analysis by Blaschko *et al*. [12] found an incidence of *de novo* ED after anterior urethroplasty of 0–38%. Xie *et al*. [1] suggested that the incidence of ED after anterior urethroplasty ranged from 16.2% to 72%.

Dogra *et al*. [13] found that mobilisation of the urethra and the possibility of injury to the cavernous nerves, bulbar artery or collateral vessels can result in ED.

In the present study, we used an IIEF-15 (EF Domain) questionnaire preoperatively, and at 3- and 6-months postoperatively for evaluation of EF. In our experience, most patients refused to be subjected to many penile Doppler US examinations due to the unpleasant feelings during the measurement. Therefore, we propose that it is possible to evaluate patients EF using IIEF-15 (EF Domain) scores alone preoperatively, and at 3 and 6 months after surgery and perform penile Doppler US at 6-months postoperatively only for patients with ED.

Of the 34 patients who underwent bulbar urethroplasty, nine had ED at 3-months postoperatively; however, six of these nine patients recovered EF by 6-months postoperatively. Therefore, there was no significant difference in the mean IIEF-15 (EF Domain) score between the preoperative score and that at 6-months postoperatively (*P* = 0.10 in the AU group and *P* = 1.0 in the SU group).

In contrast to the above mentioned data, Sangkum *et al*. [14] suggested that patients with urethral stricture and co-existing ED can be improved after urethroplasty because inflammation, oedema and presence of suprapubic tube are possible causes of transient ED, so excision of scar and fibrotic tissues during surgery and elimination of the suprapubic tube after surgery can improve EF of most patients by 6-months postoperatively, as removal of the suprapubic catheter will improve psychological state of the patient and excision of fibrous tissue will decompress the nerve supply to erectile tissues.

Because different procedures can be selected to treat patients with bulbar urethral stricture, it is necessary to compare the results of the different types of bulbar urethroplasty as regard patient EF. Mundy [15] showed that the permanent ED rate was only 5% in patients who underwent AU. Barbagli *et al*. [16] retrospectively evaluated 153 patients with bulbar urethral stricture who underwent AU using the end-to-end anastomotic technique; at a mean follow-up of >5 years the incidence of ED was 0%. Haines and Rourke [17] reported that 18 patients (20.7%) developed ED after anterior AU, while 15 patients of the 18 patients (17.2%) experienced an improvement in EF and they stated that the IIEF-5 scores remained significantly unchanged (20.16 vs 20.14, *P* = 0.98).

Hosseini *et al*. [18] reported that anastomotic urethroplasty does not significantly affect EF according to findings of the IIEF-5 questionnaire and penile duplex US. They found that the mean (SD) EF score at 3 months after urethroplasty was 13.12 (5.38) and at 6 months was 13.40 (5.53); and the differences were not statistically significant.

In our present study, 21 patients underwent AU and EF was assessed using the IIEF-15 (EF Domain) questionnaire. In the AU group, seven patients had ED at 3-months postoperatively. However, four patients regained their EF and only three patients (8.8%) had persistent ED at 6-months postoperatively, and there was a statistically significant difference between the mean IIEF-15 score preoperatively, at 3- and at 6-months postoperatively (*P* = 0.005). When comparing preoperative EF by IIEF-15 (EF Domain) score and 6-months postoperatively, there is no statistical significant difference between them (*P* = 0.1)

Reports on EF after SU differ. Dubey *et al*. [19] reported that the ED rate after flap or graft urethral reconstruction was <8%. Al-Qudah and Santucci [20] reported that no patients had ED after onlay BMG SU. Johnson and Latini [21] reported that the incidence of ED after urethral graft reconstruction ranged from 0% to 3%. For ventral BMG bulbar urethroplasty, Palminteri *et al*. [22] in a study of 52 patients reported no patients with ED postoperatively and 35% of patients had improved EF postoperatively (*P* < 0.001).

In our present study, 13 patients underwent SU and two had ED at 3 months after bulbar graft urethroplasty and these two patients regained their EF at 6-months postoperatively. None of the patients reported ED after penile-flap urethroplasty. There was no statistically significant difference between mean IIEF-15 score preoperatively, and at 3- and 6-months postoperatively (*P*= 0.13), when comparing EF by IIEF-15 (EF Domain) score between preoperatively and 6-months postoperatively, there was no statistical significant difference (*P* = 1.0). Postoperative tissue oedema and inflammation are responsible for ED observed during the first 3 months after surgery, because of impairment of the cavernous nerve fibres. With gradual subsiding of oedema and inflammation, EF recovers gradually over time, so at 6-months postoperatively, there was no significant statistical impact of bulbar urethroplasty on EF.

Beysens *et al*. [23] compared 31 patients who underwent AU with 16 patients who underwent free-graft urethroplasty based on IIEF-5 score, and found that there was a significant decrease in IIEF-5 score overall at 6-weeks follow-up (*P* = 0.026), but the decrease was significant in the AU group (*P* = 0.005) and not significant in the free-graft group. While at 6-months follow-up, there was no significant decrease in IIEF-5 score overall (*P* = 0.907) or in each group separately, reflecting the improvement in EF at 6 months after urethroplasty.

In our present study, at 3-months postoperatively, the mean (SD) IIEF-15 (EF Domain) score in AU group was 24.4 (5.6), which was statistically insignificantly different to that in SU group where it was 26.6 (2.59) (*P* = 0.60); and at 6-months postoperatively, the mean (SD) IIEF-15 score in AU group was 25.6 (5.3) and this was not statistically different to that in the SU group where it was 27.2 (1.64) (*P* = 0.87).

Some urologists reasoned that aggressive dissection and excessive use of cautery on the bulbar urethra can cause damage to the neural structures and significantly increase the incidence of ED after surgery [24,25]. So, we avoided aggressive dissection and excessive cauterisation in our present series to decrease the incidence of ED after urethroplasty.

Finally, we acknowledge some limitations to our present study, in that there were a limited number of patients, a short postoperative follow-up period, and a lack of randomisation in the SU group. So, the study necessitates confirming the results on a larger scale with more patients and longer follow-up periods.

## Conclusion

Bulbar urethroplasty either AU or SU does not affect EF significantly at 6-months follow-up.
